# Reporting of health-related quality of life in emergency laparotomy trials: a systematic review and narrative synthesis

**DOI:** 10.1007/s11136-023-03531-w

**Published:** 2023-10-26

**Authors:** Candice L. Downey, J. Lessing, D. G. Jayne

**Affiliations:** 1 Leeds Institute of Medical Research at St James’s, St. James’s University Hospital, University of Leeds, Level 7, Clinical Sciences Building, Leeds, LS9 7TF UK; 2 Leeds Teaching Hospital Trust, Beckett Street, Leeds, LS9 7TF UK

**Keywords:** Emergency, Laparotomy, Quality of life, Surgery

## Abstract

**Purpose:**

Emergency laparotomy is associated with high morbidity for the surgical patient. Understanding patients’ health-related quality of life after their surgery is important to enhance the informed consent process, and to enable the evaluation and improvement of surgical care. This review aims to summarise the use of health-related quality of life tools in clinical trials involving patients undergoing emergency laparotomy.

**Methods:**

A systematic review was undertaken of the scientific literature published in the MEDLINE^®^ and PubMed databases between January 2011 and July 2021. A narrative synthesis approach was chosen to synthesise the diverse range of studies in a structured manner. All included papers were evaluated using the Cochrane Collaboration’s tool for assessing risk of bias.

**Results:**

Eleven studies were selected for inclusion. Most of the studies had a low risk of bias. Two of the studies used health-related quality of life as the primary outcome measure. A variety of health-related quality of life measurement tools were used; the EQ-5D tool was the most popular questionnaire. Protocol adherence was dependent on the length of time which had elapsed after emergency surgery.

**Conclusion:**

There are many perceived challenges to collecting health-related quality of life data in the emergency surgery setting. Many of these can be offset with progressive trial designs. There is a need for further research in the systematic development of patient-reported outcomes for use in emergency surgery.

## Plain English summary

Emergency surgery is high-risk, and none more so than emergency laparotomy. This operation involves opening the abdomen to allow the surgeon to view and repair the organs inside. One-in-ten patients die after the surgery, and its complications can have long-term negative effects on patients’ quality of life. It’s important that the right decisions are made to reduce these effects. One way of finding out which decisions are the most helpful for patients is to measure their quality of life before and after surgery. The decisions which lead to better quality of life can then be chosen for future patients. Information about quality of life can also be used to help patients decide whether they want surgery in the first place. It can be difficult to do research in emergency laparotomy because patients are often very unwell. The aim of this research was to find out whether it is possible to measure the quality of life of patients who are having emergency laparotomy. By looking at the research published over the last ten years, we can find out how best to measure quality of life. Eleven studies were looked at, and it seems that collecting quality of life information is possible but it can be difficult for researchers to follow-up with the patients after their hospital stay. This project will now be used to improve how researchers test quality of life, to help improve the results for all patients having emergency surgery in the future.

## Introduction

In England, 40% of National Health Service (NHS) hospital admissions and 18% of surgical procedures are emergencies [1]. In 2016/2017, there were 116,000 (6%) emergency operations performed by general surgeons for digestive tract conditions, excluding appendicectomy [2]. Emergency procedures have a higher postoperative morbidity and mortality than elective procedures [3].

The emergency setting has historically been neglected by surgical researchers due to challenges in recruitment and data collection. This is particularly evident in the reporting of patient-reported outcome measures (PROMs) such as health-related quality of life (HRQoL). It has been suggested that the assessment of PROMs in the emergency setting is challenging because patients are often acutely unwell, which may affect their ability to complete questionnaires before and after surgery [4]. This is most relevant in the context of emergency laparotomy, which is performed for more urgent conditions and has high associated morbidity.

Understanding patients’ health-related quality of life after emergency laparotomy is important to enable the evaluation of surgical care and to improve standards. The NHS is a healthcare system with finite resources. Fixed budgets mean that decisions about new treatments cannot be made on the basis of clinical effectiveness alone; new interventions must be shown to be cost-effective before they can be widely adopted. The NICE Reference Case recommends that the calculation of the cost-effectiveness of an intervention should include quality of life measures [5]. The collection of a standardised set of PROMs can enable comparisons between interventions and providers to stimulate improvements in services [6]. This is important in the emergency laparotomy setting where there is no standardised core outcome set, yet the patients are a heterogeneous group with a range of surgical pathologies and exposed to a variety of clinical care processes [7]. Evidence obtained from quality of life studies can also help inform shared decision-making before undertaking potentially high-risk surgery.

An earlier review by Stevens et al*.* summarised the collection of PROM data in randomised controlled trials (RCTs) in unplanned general surgery up to 2011 and identified only two RCTs which collected health-related quality of life data [4] after emergency laparotomy. This review aims to update this work in light of recent increased interest in the field, summarise the HRQoL tools that are commonly used in emergency laparotomy trials and, in doing so, discover the feasibility of collecting health-related quality of life data in this setting.

## Materials and methods

### Study design

A systematic review methodology was adopted for the study, employing the principles and methods provided by the Centre for Reviews and Dissemination guidelines and following the PRISMA statement [8]. A narrative synthesis approach was chosen to synthesise the diverse range of selected studies in a structured manner, following the European Social Research Council Guidance on the Conduct of Narrative Synthesis in Systematic Reviews [9].

### Search strategy

A systematic review of the scientific literature was performed by the first author. MEDLINE^®^ and PubMed were searched for articles published from January 2011 to July 2021. The earlier date was chosen as this was the upper limit of the previous review’s search strategy.

In order to extract all available data regarding health-related quality of life after emergency laparotomy, the search strategy was kept necessarily broad. The search strategy was devised with the help of a Research Support Advisor at the Leeds University Library, using both MeSH and/or keyword search terms according to the database.

The search strategy for MEDLINE^®^ is detailed below and further details can be provided on request from the authors.

1. “acute” OR "emergency" OR "unplanned" OR "urgent" OR "trauma"
2. “laparotomy” OR “surger*” OR “surgic*” OR “operation”
3. “trial”
4. “randomi*”
5. #3 OR #4
6. #1 AND #2 AND #5

In addition, citations and reference lists of selected studies were reviewed to identify any missed papers.

### Identifying relevant papers

Publications were selected in two phases: first by review of title and abstract and then by full text review.

Studies were selected if they included adult human subjects undergoing open emergency general surgery. Selection was limited to peer-reviewed publications of clinical trials. Cohort studies, consensus papers and protocols were excluded as they did not support the research question. Study selection was not limited by the type of surgery or the outcomes measured. Papers had to be written in English due to lack of translation resources. Studies regarding the paediatric population were excluded, as were studies describing the development or validation of surgical techniques or equipment.

Selected papers were then added to the two relevant papers from the previous review.

### Data extraction and analysis

A narrative synthesis approach was chosen to synthesise the diverse range of studies in a structured manner, following the European Social Research Council Guidance on the Conduct of Narrative Synthesis in Systematic Reviews [9]. Briefly, studies were tabulated and grouped by the year of publication, the HRQoL tools used and the study population. Patterns were identified and the evidence was synthesised to provide a meaningful narrative, relevant to the research question.

### Quality assessment

All included papers were evaluated using the Cochrane Collaboration’s tool for assessing risk of bias [10]. This assesses bias according to key domains, including selection, detection, attrition and reporting bias, which are evaluated within the specific context of each study. All manuscripts were assessed independently by two of the authors, with discrepancies resolved through consensus.

## Results

**Fig. 1:**
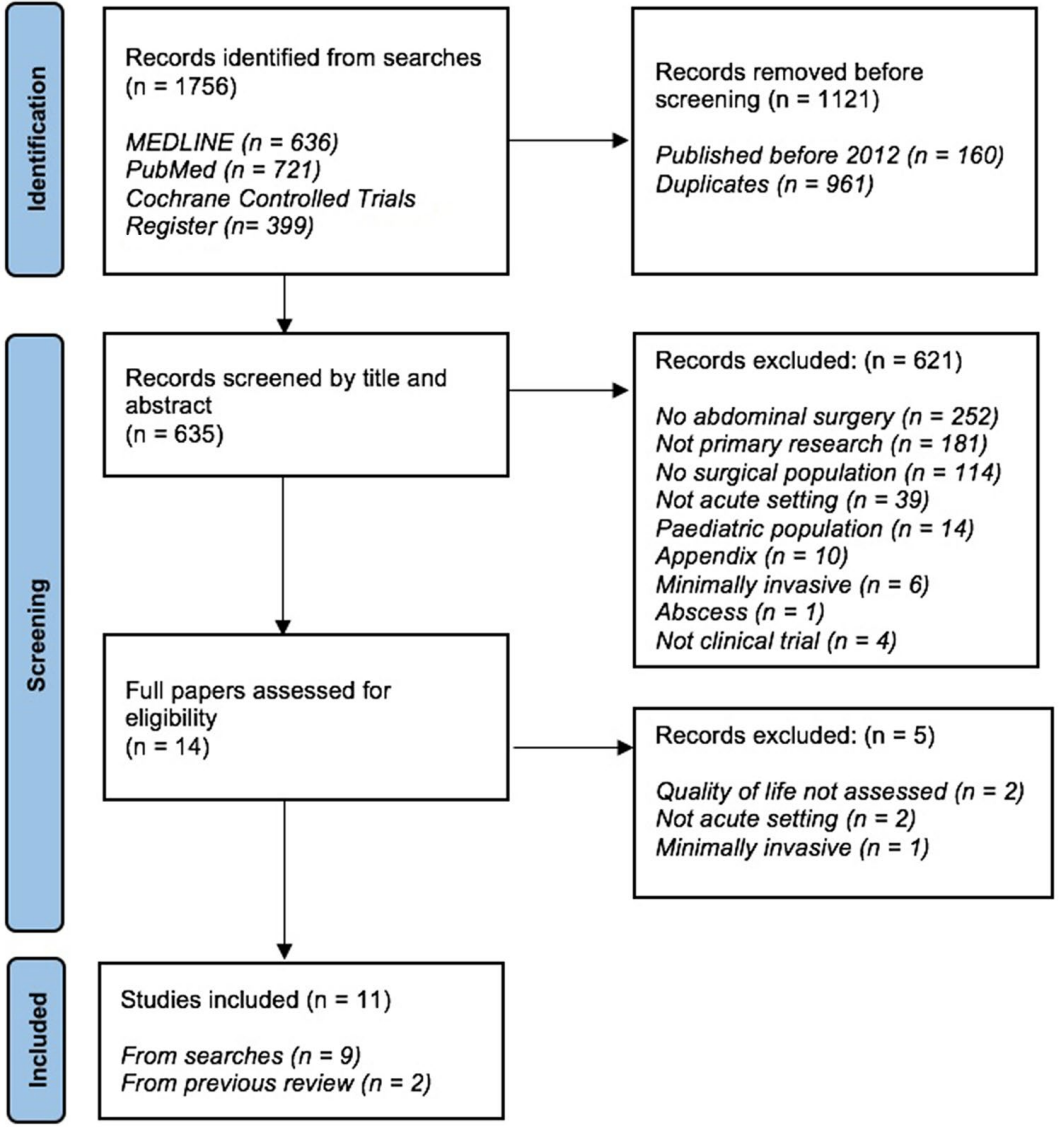
PRISMA diagram summarising selection process

The search identified 1756 papers. Duplicates were eliminated. Eleven papers met the inclusion criteria: nine from the literature search; two from the earlier review. A flow diagram of the selection process is shown in Fig. 1.

### Study demographics

The included papers were published between 2011 and 2020. Two papers were publications of the long-term follow up from two other included studies, as detailed in the summary in Table 1. The patient populations included those with malignant left-sided bowel obstruction, gallbladder pathologies, abdominal aortic aneurysm, acute perforated diverticulitis and infected necrotizing pancreatitis. Comparators to open emergency surgery included laparoscopic procedures (*n* = 6), colonic stenting (*n* = 1) and endovascular procedures (*n* = 1). Other interventions under investigation included a mechanical anti-adhesion barrier (*n* = 1), a graded approach to the management of necrotizing pancreatitis (*n* = 1) and a perioperative quality improvement programme (*n* = 1).

**Table 1:** Summary of included papers

First author, date, country	Surgery/surgical condition	Intervention (number of participants)	Comparator (number of participants)	Primary outcome	HRQoL tool/s used	Baseline HRQoL assessed	Timepoints HRQoL assessed	Findings	Risk of bias
van der Wal, 2011, Netherlands [17]	Hartmann’s procedure	Mechanical anti-adhesion barrier (*n* = 16)	Usual care (*n* = 19)	Incidence of abdominal complaints	SF-36^a^ EQ-5D^b^	No	11–13 years after participation	No difference in HRQol between groups	Some concerns
van Hooft, 2011, Netherlands [13]	Acute left-sided malignant colonic obstruction	Colonic stenting (*n* = 47)	Emergency surgery (*n* = 51)	Mean global health status	EORTC QLQ-C30^c^	Yes	4, 12 and 24 weeks after inclusion	No difference in health status between groups**	Low
Rosenmuller*, 2013, Sweden [18]	Cholecystectomy (30% acute)	Small incision open procedure (*n* = 172)	Laparoscopic procedure (*n* = 183)	Costs and quality of life	EQ-5D	Yes	3, 7, 11 and 30 days after surgery	Quality of life significantly lower in open group	Some concerns
Kapma, 2014, Netherlands [19]	Ruptured abdominal aortic aneurysm	Endovascular repair (*n* = 57)	Open repair (*n* = 59)	Cost-effectiveness	SF-36 EQ-5D	No	30 days, 3 months and 6 months after surgery	No difference in HRQol between groups	Low
Schultz*, 2015, Norway and Sweden [20]	Acute perforated diverticulitis	Laparoscopic lavage (*n* = 101)	Primary colonic resection (*n* = 98)	Major complications	Cleveland QOL instrument	No	90 days after surgery	No difference in HRQol between groups	Low
Thornell, 2016, Sweden and Denmark [21]	Perforated diverticulitis	Laparoscopic lavage (*n* = 43)	Hartmann’s procedure (*n* = 40)	Reoperation rate within 1 year	SF-36 EQ-5D	No	At discharge, and 6 and 12 months after discharge	No difference in HRQol between groups	Low
Rosenmuller*, 2017, Sweden [22]	Cholecystectomy (30% acute)	Small incision open procedure (*n* = 151)	Laparoscopic procedure (*n* = 172)	Costs and quality of life	EQ-5D	Yes	3, 7, 11 and 30 days and 12 months after surgery	No difference in HRQol between groups	Low
Schultz*, 2017, Norway and Sweden [23]	Acute perforated diverticulitis	Laparoscopic lavage (*n* = 101)	Primary colonic resection (*n* = 98)	Major complications	Cleveland QOL instrument	No	1 year after surgery	No difference in HRQol between groups	Low
Yang, 2019, United Kingdom [11]	Emergency abdominal surgery	Perioperative quality improvement programme (*n* = 7,374)	Usual care (*n* = 8,482)	Cost-effectiveness	EQ-5D	Yes	90 and 180 days after surgery	No difference in HRQol between groups	Low
Hollemans, 2019, Netherlands [24]	Infected necrotizing pancreatitis	Step-up approach (*n* = 30)	Primary open necrosectomy (*n* = 36)	Composite of death or major complications	SF-36 EQ-5D	No	3, 6, and 12 months, and 5 years after discharge	No difference in HRQol between groups	Low
Harji, 2020, United Kingdom [12]	Emergency abdominal surgery	Laparoscopic surgery (*n* = 33)	Open surgery (*n* = 31)	Feasibility	SF-12^d^ EQ-5D GIQLI^e^	Yes	3, 7 and 30 days, and 3, 6 and 12 months after surgery	Data compliance reduced over time	Low

^*^Same trial, short- and long-term results published separately

^**^Trial stopped early due to safety concerns

^a^SF-36 Medical Outcome Study Short Form-36

^b^EQ-5D EuroQol-5 Dimensions

^c^EORTC QLQ-C30 European Organization for Research and Treatment of Cancer quality of life questionnaire

^d^SF-12 Medical Outcome Study Short Form-12

^e^GIQLI Gastrointestinal Quality of Life Index

### HRQoL tools used

Five of the studies used only one HRQoL tool; six used two or more tools. The most commonly used HRQoL tool was the EuroQol-5 Dimensions (EQ-5D) score (*n* = 8). The Medical Outcome Study Short Form-36 (SF-36) was used in four studies. The Medical Outcome Study Short Form-12 (SF-12) was used in one study. Other HRQoL tools included the European Organization for Research and Treatment of Cancer quality of life questionnaire (EORTC QLQ-C30) (*n* = 1), the Gastrointestinal Quality of Life Index (GIQLI) (*n* = 1) and the Cleveland Quality of Life Instrument (*n* = 2).

### Feasibility of HRQoL collection

Two of the studies used HRQoL as the primary outcome measure; one of these studies was terminated early due to safety concerns about the intervention. Five of the included studies collected HRQoL data at baseline. HRQoL data were collected at different timepoints in each study, ranging from three days to 13 years post-surgery. The most common timepoint at which data were collected was 3 months after surgery. The adherence to protocol decreased if the time from surgery to survey was longer; for instance, Yang et al*.* had 19% missing data at 90 days and 24% missing data at 180 days, compared with 1% missing data at baseline [11]. Harji et al*.* found that overall HRQoL questionnaire compliance dropped from 98% on Day 3 to 58% at 12 months after surgery [12]; interviews with patients identified that they perceived the burden of questionnaire completion to be too high and that questions were irrelevant to their clinical status (the study used EQ-5D, SF-12 and GIQLI tools). Inpatient questionnaire compliance was higher than outpatient compliance, and telephone follow-up yielded lower compliance than face-to-face follow-up.

### Quality assessment

Nine of the studies were scored as ‘Low’ on the risk of bias assessments; two studies were scored as ‘Some concerns’ due to baseline differences between the two intervention groups. In general, studies showed good compliance with completion of reporting and the recruited participants demonstrated good representation of the patient population. The most common source of potential bias in the studies was the lack of blinding of participants to the intervention they received, explained by one paper comparing colonic stenting to surgery as due to ‘the obvious strategies under assessment’ [13]. One study did attempt to blind participants to their allocation to either laparoscopic or open surgery, but found that ‘patients found the process of blinding unnecessary, and often guessed their treatment allocation correctly’ [12]. Another common source of potential bias was deviations from the trial protocol, especially when not balanced between trial arms.

## Discussion

This review summarises the use of health-related quality of life tools in clinical trials involving patients having emergency laparotomy procedures. An earlier review identified only two studies which measured HRQoL in this patient group between 2007 and 2012. This work updates these findings to include a further 9 studies which have been published up to 2021. Only two of the studies used HRQoL as the primary outcome measure. A variety of HRQoL tools were used, with EQ-5D the most popular questionnaire. Protocol adherence was dependent on the length of time which had elapsed after emergency surgery.

The emergency setting has historically been neglected by surgical researchers, but more recently there has been increased interest in this field. In 2011, the Department of Health and the Royal College of Surgeons of England published ‘The Higher Risk General Surgical Patient’ report, which found that the care of patients requiring emergency surgical management is frequently disjointed, protracted and not always patient centred, and recommended that a national audit of outcomes should be conducted for adult patients undergoing unscheduled general surgery [14]. The National Emergency Laparotomy Audit (NELA) commenced data collection in 2013 and have since published six Patient Audit Reports into the care of patients undergoing emergency laparotomy in England and Wales. NELA is limited, however, by the constraint of only being able to collect data linked to existing standards of care, and none of its source data are derived from randomised controlled studies [7]. Patient outcomes are limited to mortality, critical care use and return to theatre rates. There is no data collection of health-related quality of life or patient-reported outcome measures. This is in contrast to national audits of elective surgery; the Perioperative Quality Improvement Programme (PQIP) is a national audit of more than 30,000 patients who have undergone planned surgery in the last 4 year and includes information on patients’ health-related quality of life before and after surgery.

There are many reasons cited for the paucity of health-related quality of life data in the emergency surgery setting. There is perceived difficulty in recruiting patients who are critically unwell. The time constraints of the emergency context bring particular challenges to the process of informed consent. The patient group is heterogenous in both baseline characteristics, surgical pathologies and clinical care needs, requiring large volumes of trial participants to ‘separate signal from noise’ [7]. The data collected by the studies included in this review, and recent large trials in the critical care setting, demonstrate that these challenges are not insurmountable. Potential solutions include less conventional research trial designs which may include post-hoc consent.

The collection of patient-reported health-related quality of life data is vital in trials in the emergency surgery setting. In addition to aiding the informed consent process, health-related quality of life data provides a common measure through which all interventions can be compared, allowing the evaluation and improvement of surgical care in a resource-limited healthcare system. Although little is known about how to optimize data collection in this setting, a recent study has examined the feasibility of collecting patient-reported outcome data during unplanned surgical hospital admissions [15]. It found that, with specific research support during the working week, good baseline response rates to questionnaires could be achieved. The waning protocol compliance found in the studies included in this review indicate the need for more relevant, patient-focussed HRQoL tools. The most popular tool, the EQ-5D questionnaire, is the most generic. None of the tools used in the selected studies are specific for surgery or emergency admissions, and thus many of the most important considerations for postoperative patients may be overlooked.

One of the important limitations of this review is this heterogeneity of patient cohorts and HRQoL tools. The results must be interpreted within the limitations of the original studies, which may limit the generalisability of the findings. Study quality was generally high, although many of the selected papers shared common limitations. Most studies were limited to small populations and follow-up periods were relatively short.

There is a need for further research in the systematic development of disease-specific PROMs for use in emergency admissions, including psychometric testing for use in emergency laparotomy [16].

Future research could focus on:

1. Identification of the outcome measures that are most valuable to key stakeholders in the emergency surgery setting, including patients.
2. The barriers to collecting patient-reported outcome measures such as health-related quality of life.
3. Collating the results of studies which include health-related quality of life measures to better inform patients in the emergency surgery setting.

## Data Availability

The data used in this review can be provided upon reasonable request to the corresponding author.
